# Conversion of 2-methyl-4-styryl­quino­lines into 2,4-distyryl­quino­lines: synthesis, and spectroscopic and structural characterization of five examples

**DOI:** 10.1107/S2053229623001432

**Published:** 2023-02-22

**Authors:** Diana R. Vera, Diana M. Ardila, Alirio Palma, Justo Cobo, Christopher Glidewell

**Affiliations:** a Laboratorio de Síntesis Orgánica, Escuela de Química, Universidad Industrial de Santander, AA 678, Bucaramanga, Colombia; b Departamento de Química Inorgánica y Orgánica, Universidad de Jaén, 23071 Jaén, Spain; c School of Chemistry, University of St Andrews, Fife, KY16 9ST, United Kingdom; University of Notre Dame, USA

**Keywords:** heterocyclic compounds, synthesis, quino­lines, styryl­quino­lines, NMR spectroscopy, crystal structure, mol­ecular conformation, hy­dro­gen bonding, supra­molecular assembly, privilaged scaffold

## Abstract

Four new 2,4-distyryl­quino­lines and one 2-styryl-4-[2-(thio­phen-2-yl)vin­yl]quinoline have been synthesized using indium trichloride condensation reactions between aromatic aldehydes and the corresponding 2-methyl­quino­lines, which were themselves prepared using Friedländer annulation reactions between mono- or diketones and (2-amino­phen­yl)chalcones: the products have all been fully characterized by spectroscopic and crystallographic methods. 2,4-Bis[(*E*)-styr­yl]quino­line, C_25_H_19_N, (IIa), and its di­chloro analogue, 2-[(*E*)-2,4-di­chloro­styr­yl]-4-[(*E*)-styr­yl]quino­line, C_25_H_17_Cl_2_N, (IIb), exhibit different orientations of the 2-styryl unit relative to the quino­line nucleus. In each of the 3-benzoyl analogues {2-[(*E*)-4-bromo­styr­yl]-4-[(*E*)-styr­yl]quinolin-3-yl}(phen­yl)methanone, C_32_H_22_BrNO, (IIc), {2-[(*E*)-4-bromo­styr­yl]-4-[(*E*)-4-chloro­styr­yl]quinolin-3-yl}(phen­yl)methanone, C_32_H_21_BrClNO, (IId), and {2-[(*E*)-4-bromo­styr­yl]-4-[(*E*)-2-(thio­phen-2-yl)vin­yl]quinolin-3-yl}(phen­yl)methanone, C_30_H_20_BrNOS, (IIe), the orientation of the 2-styryl unit is similar to that in (IIa), but the orientation of the 4-aryl­vinyl units show considerable variation. The thio­phene unit in (IIe) is disordered over two sets of atomic sites having occupancies of 0.926 (3) and 0.074 (3). There are no hy­dro­gen bonds of any kind in the structure of (IIa), but in (IId), a single C—H⋯O hy­dro­gen bond links the mol­ecules into cyclic centrosymmetric *R*_2_^2^(20) dimers. A combination of C—H⋯N and C—H⋯π hy­dro­gen bonds links the mol­ecules of (IIb) into a three-dimensional framework structure. A combination of three C—H⋯π hy­dro­gen bonds links the mol­ecules of (IIc) into sheets, and a combination of C—H⋯O and C—H⋯π hy­dro­gen bonds forms sheets in (IIe). Comparisons are made with the structures of some related compounds.

## Abstract 2

Five new 4-(aryl­vin­yl)-2-styrylquino­lines have been synthesized using indium chloride-catalyzed condensation reactions between their 4-(aryl­vin­yl)-2-methyl- analogues and either mono- or diketones. The supra­molecular arrangements range from isolated mol­ecules *via* hy­dro­gen-bonded dimers and sheets to three-dimensional framework structures.

## Introduction

The quino­line nucleus is considered to be one of the most privileged scaffolds and to be a crucial pharmacophore in drug discovery because of its occurrence in a wide variety of natural and synthetic biologically active mol­ecules (Solomon & Lee, 2011 ▸; Musiol *et al.*, 2017 ▸; Matada *et al.*, 2021 ▸). The outstanding therapeutic importance of quino­line derivatives is well known, particularly in the treatment of, for example, microbial (Lam *et al.*, 2014 ▸; Zhang *et al.*, 2018 ▸), malarial (Kaur *et al.*, 2010 ▸; Hu *et al.*, 2017 ▸; Okombo & Chibale, 2018 ▸; Orozco *et al.*, 2020 ▸), fungal (Musiol *et al.*, 2010 ▸; Kumar *et al.*, 2011 ▸), inflammatory (Chen *et al.*, 2006 ▸; Gilbert *et al.*, 2008 ▸), viral (Ghosh *et al.*, 2008 ▸; Matada *et al.*, 2021 ▸), protozoal (Fakhfakh *et al.*, 2003 ▸; Franck *et al.*, 2004 ▸; Kumar *et al.*, 2009 ▸), cardiovascular (Cai *et al.*, 2007 ▸; Bernotas *et al.*, 2009 ▸) and neoplastic diseases (Afzal *et al.*, 2015 ▸; Musiol, 2017 ▸; Cortes *et al.*, 2018 ▸; Lauria *et al.*, 2021 ▸; Yadav & Kamal, 2021 ▸).

Among different classes of quino­line derivatives, styryl­quino­lines, especially 2-styryl­quino­lines and to a lesser extent 4-styryl­quino­lines, have been studied extensively, mainly because of their potential as inhibitors of HIV-1 integrase (Leonard & Roy, 2008 ▸; Mahajan *et al.*, 2018 ▸; Mousnier *et al.*, 2004 ▸) and as anti­microbial (Kamal *et al.*, 2015 ▸), anti­fungal (Cieslik *et al.*, 2012 ▸; Szczepaniak *et al.*, 2017 ▸), anti-asthma (Matada *et al.*, 2021 ▸) and anti­cancer agents (Chang *et al.*, 2010 ▸; Mrozek-Wilczkiewicz *et al.*, 2015 ▸, 2019 ▸). The pharmacological 
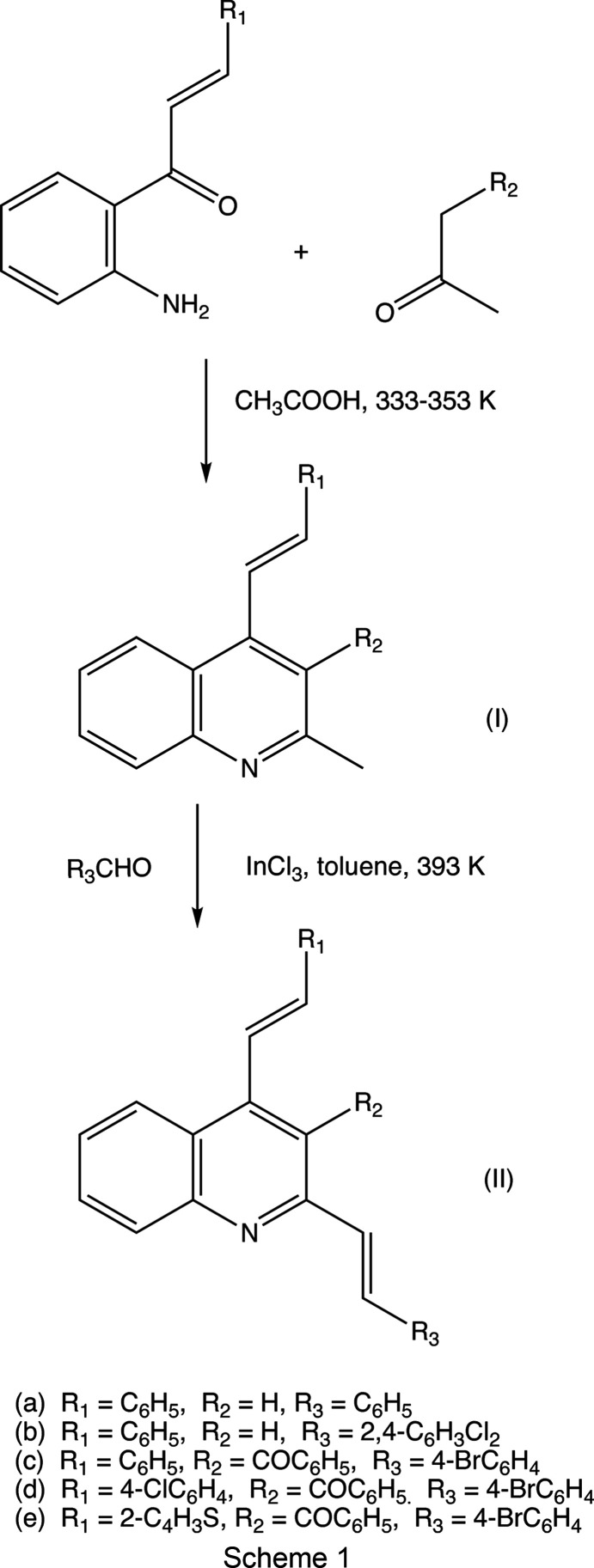
 relevance of these types of quino­line derivatives has prompted the development of different methodologies for the synthesis of drug-like compounds containing such styryl­quino­line scaffolds (Staderine *et al.*, 2011 ▸; Yaragorla *et al.*, 2015 ▸; Sharma *et al.*, 2017 ▸; Musiol, 2020 ▸; Hazra *et al.*, 2020 ▸; Zhang *et al.*, 2020 ▸; Li *et al.*, 2021 ▸; Omar & Hormi, 2009 ▸; Lee *et al.*, 2009 ▸ Alacíd & Nájera, 2009 ▸; Jamal & Teo, 2014 ▸; Jamal *et al.*, 2016 ▸; Satish *et al.*, 2019 ▸; Meléndez *et al.*, 2020 ▸).

Unlike 2-styryl- and 4-styryl­quino­lines, the closely-related 2,4-distyryl­quino­lines have been scarcely investigated, with very few publications related to their synthesis and biological evaluation, and this scarcity may be due, at least in part, to the lack of satisfactory methods for their synthesis. The few re­ported 2,4-distyryl­quino­lines have been prepared by methods such as one-pot successive Arbuzov/Horner–Wadsworth–Emmons reactions using ethyl 4-(bromo­meth­yl)-2-(chloro­meth­yl)quino­line-3-carboxyl­ate as the key precursor (Gao *et al.*, 2018 ▸), and the Knoevenagel-type condensation of 2-methyl-4-styryl­quino­line with aromatic aldehydes, catalysed by sodium acetate (Satish *et al.*, 2019 ▸).

We have recently described an efficient and straightforward synthetic pathway, based on Friedländer annulation and starting from readily available 1-(2-amino­phen­yl)-3-aryl­prop-2-en-1-ones, to obtain several new series of polysubstituted 2-methyl-4-styryl­quino­lines (Meléndez *et al.*, 2020 ▸; Vera *et al.*, 2022 ▸). In an expansion of the scope of this route, in respect of both utility and flexibility, we now describe the synthesis, spectroscopic characterization, and mol­ecular and supra­molecular structures of a matched set of five closely-related 2,4-distyryl­quino­lines, namely, 2,4-bis­[(*E*)-styr­yl]quino­line, (IIa), 2-[(*E*)-2,4-di­chloro­styr­yl]-4-[(*E*)-styr­yl]quino­line, (IIb), {2-[(*E*)-4-bromo­styr­yl]-4-[(*E*)-styr­yl]quinolin-3-yl}(phen­yl)methanone, (IIc), {2-[(*E*)-4-bromo­styr­yl]-4-[(*E*)-4-chloro­styr­yl]quinolin-3-yl}(phen­yl)methanone, (IId), and {2-[(*E*)-4-bromo­styr­yl]-4-[(*E*)-2-(thio­phen-2-yl)vin­yl]quinolin-3-yl}(phen­yl)methanone, (IIe) (see Scheme 1), which differ only in the nature of the substituents at position C3 of the quino­line ring and the substituents in the 4-(aryl­vin­yl) fragments. To the best of our knowledge, these 2,4-distyryl­quino­lines have not been reported previously.

The work reported here can be regarded as a continuation of an earlier crystallographic study which reported the structures of 4-styryl­quino­lines having different substituents at the C2 and C3 positions (Rodríguez *et al.*, 2020 ▸; Vera *et al.*, 2022 ▸; Ardila *et al.*, 2022 ▸).

## Experimental

### Synthesis and crystallization

For the synthesis of compounds (IIa)–(IIe), a mixture of the appropriate 2-methyl-4-styryl­quino­line, (I) (see Scheme 1), prepared as described previously (Meléndez *et al.*, 2020 ▸; Vera *et al.*, 2022 ▸) (1.0 mmol), the appropriate aromatic aldehyde (4.0 mmol) and indium trichloride (10 mmol%) in dry toluene (1.2 ml) was stirred magnetically and heated at 393 K until the reactions were complete, shown by the complete consumption of (I), as monitored by thin-layer chromatography (TLC). The reaction times for completion were 18 h for (IIa), 16 h for (IIb), 17 h for (IIc) and 21 h for both (IId) and (IIe). Each reaction mixture was then allowed to cool to ambient tem­pe­ra­ture, washed with chloro­form and the resulting suspension was removed by filtration before the filtrate was con­cen­trated under reduced pressure. In each case, the resulting crude product was purified by silica-gel column chromatography using hepta­ne–ethyl acetate mixtures as eluent (compositions ranged from 10:1 to 2:1 *v*/*v*) to give the required solid products (IIa)–(IIe). Crystallization from ethyl acetate–heptane, at ambient tem­pe­ra­ture and in the presence of air, gave crystals suitable for single-crystal X-ray diffraction.

In the NMR data listed below, unprimed ring atoms form part of the quino­line units; ring atoms carrying a single prime form part of the styryl units attached at position C2 of the quino­line system; ring atoms carrying double primes form part of the styryl units attached at position C4 for compounds (IIa) and (IIb), or part of the benzoyl units attached at position C3 for compounds (IIc)–(IIe); and ring atoms carrying triple primes form part of the styryl units attached at position C4 for compounds (IIc)–(IIe).

2,4-Bis[(*E*)-styr­yl]quino­line, (IIa). Yellow solid, yield 0.11 g (71%), m.p. 393–394 K, *R*_F_ = 0.40 (21% ethyl acetate–hepta­ne). IR (ATR, cm^−1^): 3019 [C(*sp* ^2^)H], 1723 (C=N), 1633 (C=C_vin­yl_), 1581 (C=C_arom_), 1541 (C=C_arom_), 961 (=C—H_*trans*_). NMR (CDCl_3_): δ(^1^H) (400 MHz) 8.15 (*d*, *J* = 8.3 Hz, 1H, H5), 8.12 (*d*, *J* = 8.4 Hz, 1H, H8), 7.84 (*d*, *J* = 1.3 Hz, 1H, H3), 7.81 (*d*, *J* = 16.1 Hz, 1H, H_A′′_C=), 7.76 (*d*, *J* = 16.0 Hz, 1H, =CH_B′_), 7.72–7.68 (*m*, 1H, H7), 7.68–7.65 (*m*, 4H, H2′, H6′, H2′′, H6′′), 7.53 (*ddt*, *J* = 8.2, 6.9, 1.3 Hz, 1H, H6), 7.47–7.43 (*m*, 1H, H_A′_C=), 7.46–7.41 (*m*, 4H, H3′, H5′, H3′′, H5′′), 7.40–7.32 (*m*, 2H, H4′, H4′′), 7.38–7.34 (*m*, 1H, =CH_B′′_); δ(^13^C) (100 MHz) 155.7 (C2), 148.8 (C8a), 143.3 (C4), 136.7 (C1′′), 136.6 (C1′), 135.0 (=CH_B′′_), 134.3 (=CH_B′_), 129.9 (C8), 129.6 (C7), 128.9 (C3′′, C5′′), 128.8 (C3′, C5′, C4′′, H_A′_C=), 128.6 (C4′), 127.3 (C2′′, C6′′), 127.2 (C2′, C6′), 126.2 (C6), 125.7 (C4a), 123.4 (C5, H_A′_—C=), 115.6 (C3). HRMS (ESI^+^) *m*/*z* found for [*M* + H]^+^ 334.1590, C_25_H_19_N requires 333.1589.

2-[(*E*)-2,4-Di­chloro­styr­yl]-4-[(*E*)-styr­yl]quino­line, (IIb). Yellow solid, yield 0.19 g (77%), m.p. 449–450 K, *R*_F_ = 0.41 (21% ethyl acetate–hepta­ne). IR (ATR, cm^−1^): 3055 [C(*sp* ^2^)H], 1639 (C=N), 1580 (C=C_vin­yl_), 1541 (C=C_arom_), 1473 (C=C_arom_), 960 (=C—H_*trans*_). NMR (CDCl_3_): δ(^1^H) (400 MHz) 8.17 (*dd*, *J* = 8.4, 1.5 Hz, 1H, 5H), 8.11 (*dd*, *J* = 8.6, 1.3 Hz, 1H, 8H), 8.03 (*d*, *J* = 16.3 Hz, 1H, =CH_B′_), 7.86 (s, 1H, 3H), 7.81 (*d*, *J* = 16.3 Hz, 1 H, H_A′′_C=), 7.75 (*d*, *J* = 8.4 Hz, 1H, H6′), 7.73 (*ddd*, *J* = 8.4, 6.9, 1.4 Hz, 1H, H7), 7.67–7.64 (*m*, 2H, H2′′, H6′′), 7.56 (*ddd*, *J* = 8.3, 6.8, 1.3 Hz, 1H, H6), 7.47–7.42 (*m*, 3H, H3′, H3′′, H5′′), 7.39 (*d*, *J* = 16.3 Hz, 1H, =CH_B′′_), 7.39–7.35 (*m*, 1H, H4′′), 7.38 (*d*, *J* = 16.3 Hz, 1H, H_A′_C=), 7.29 (*dd*, *J* = 8.2, 2.0 Hz, 1H, H5′); δ(^13^C) (100 MHz) 155.1 (C2), 148.8 (C8a), 143.5 (C4), 136.6 (C1′′), 135.3 (=CH_B′′_), 134.6 (C2′), 134.5 (C4′), 133.4 (C1′), 132.3 (H_A′_C=), 130.1 (C8), 129.8 (C7, C3′), 129.0 (C3′′, C5′′), 128.9 (=CH_B′_), 128.8 (C4′′), 127.7 (C6′), 127.5 (C5′), 127.2 (C2′′, C6′′), 126.5 (C6), 125.8 (C4a), 123.5 (C5), 123.2 (H_A′′_C=), 115.4 (C3). HRMS (ESI^+^) *m*/*z* found for [*M* + H]^+^ 402.0812, C_25_H_17_^35^Cl_2_N requires 402.0811.

{2-[(*E*)-4-Bromo­styr­yl]-4-[(*E*)-styr­yl]quinolin-3-yl}(phen­yl)methanone, (IIc). Orange solid. yield 0.20 g (86%), m.p. 471–472 K, *R*_F_ = 0.38 (9.1% ethyl acetate–hepta­ne). IR (ATR, cm^−1^): 3025 [C(*sp* ^2^)H], 1666 (C=O), 1627 (C=N), 1595 (C=C_vin­yl_), 1535 (C=C_arom_), 1483 (C=C_arom_), 957 (=C—H_*trans*_). NMR (CDCl_3_): δ(^1^H) (400 MHz) 8.20 (*dd*, *J* = 8.5, 1.2 Hz, 1H, H8), 8.12 (*dd*, *J* = 8.4, 1.4 Hz, 1H, H5), 8.00 (*d*, *J* = 15.5 Hz, 1H, =CH_B′_), 7.80 (*ddd*, *J* = 8.6, 6.9, 1.3 Hz 1H, H7), 7.78–7.76 (*m*, 2H, H2′′, H6′′), 7.57 (*ddd*, *J* = 8.3, 6.9, 1.2 Hz, 1H, H6), 7.56–7.52 (*m*, 1H, H4′′), 7.44–7.42 (*m*, 2H, H3′, H5′), 7.39 (*t*, *J* = 7.8 Hz, 2H, H3′′, H5′′), 7.35–7.32 (*m*, 2H, H2′, H6′), 7.30–7.26 (*m*, 5H, H2′′′, H6′′′, H3′′′, H5′′′, H4′′′), 7.23 (*dd*, *J* = 16.4, 0.8 Hz, 1H, H_A′′′_C=), 7.09 (*dd*, *J* = 15.5, 0.8 Hz, 1H, H_a′_C=), 6.87 (*d*, *J* = 16.4 Hz, 1H, =CH_B′′′_); δ(^13^C) (100 MHz) 198.4 (C=O), 151.4 (C2), 148.1 (C8a), 142.4 (C4), 139.5 (=CH_B′′′_), 137.9 (C1′′), 136.3 (C1′′′), 135.4 (C1′), 135.0 (=CH_B′_), 134.0 (C4′′), 131.8 (C3′, C5′), 131.1 (C3), 130.6 (C7), 130.0 (C8), 129.5 (C2′′, C6′′), 129.0 (C2′, C6′), 128.9 (C3′′, C5′′), 128.8 (C4′′′), 128.7 (C3′′′, C5′′′), 126.9 (C6), 126.8 (C2′′′, C6′′′), 125.4 (C4a), 125.2 (C5), 125.0 (H_A′_C=), 122.7 (C4′), 122.1 (H_A′′_C=). HRMS (ESI^+^) *m*/*z* found for [*M* + H]^+^ 516.09656, C_32_H_22_^79^BrNO requires 516.09575.

{2-[(*E*)-4-Bromo­styr­yl]-4-[(*E*)-4-chloro­styr­yl]quinolin-3-yl}(phen­yl)methanone, (IId). Orange solid, yield 0.19 g (80%), m.p. 476–477 K, *R*_F_ = 0.35 (9.1% ethyl acetate–hepta­ne). IR (ATR, cm^−1^): 3046 [C(*sp* ^2^)H], 1661 (C=O), 1630 (C=N), 1590, 1595 (C=C_vin­yl_), 1540 (C=C_arom_), 1487 (C=C_arom_), 978 (=C—H_*trans*_). NMR (CDCl_3_): δ(^1^H) 8.20 (400 MHz) (*dd*, *J* = 8.4, 1.4 Hz, 1H, H8), 8.08 (*ddd*, *J* = 8.4, 1.4, 0.7 Hz, 1H, H5), 8.00 (*d*, *J* = 15.5 Hz, 1H, =CH_B′_), 7.80 (*ddd*, *J* = 8.4, 6.8, 1.3 Hz, 1H, H7), 7.76–7.74 (*m*, 2H, H2′′, H6′′), 7.57 (*ddd*, *J* = 8.3, 6.8, 1.3 Hz, 1H, H6), 7.56–7.52 (*m*, 1H, H4′′), 7.44–7.42 (*m*, 2H, H3′, H5′), 7.39 (*t*, *J* = 7.8 Hz, 2H, H3′′, H5′′), 7.34–7.31 (*m*, 2H, H2′, H6′), 7.27–7.25 (*m*, 2H, H3′′′, H5′′′), 7.22–7.18 (*m*, 2H, H2′′′, H6′′′), 7.20 (*d*, *J* = 16.4 Hz, 1H, H_A′′′_C=), 7.08 (*d*, *J* = 15.5 Hz, 1H, H_A′_C=), 6.81 (*d*, *J* = 16.4 Hz, 1H, =CH_B′′′_); δ(^13^C) (100 MHz) 198.4 (C=O), 151.4 (C2), 148.1 (C8a), 142.0 (C4), 138.1 (=CH_B′′′_), 137.9 (C1′′), 135.4 (C1′), 135.1 (=CH_B′_), 134.7 (C4′′′), 134.6 (C1′′′), 134.1 (C4′′), 131.8 (C3′, C5′), 131.1 (C3), 130.7 (C7), 130.0 (C8), 129.5 (C2′′, C6′′), 129.0 (C2′, C6′, C3′′′, C5′′′), 128.9 (C3′′, C5′′), 128.0 (C2′′′, C6′′′), 127.0 (C6), 125.2 (C4a), 125.1 (HH_A′_C=), 124.9 (C5), 122.7 (C4′, H_A′′′_C=). HRMS (ESI^+^) *m*/*z* found for [*M* + H]^+^ 550.05750, C_32_H_21_^79^Br^35^ClNO requires 550.05678.

{2-[(*E*)-4-Bromo­styr­yl]-4-[(*E*)-2-(thio­phen-2-yl)vin­yl]quino­lin-3-yl}(phen­yl)methanone, (IIe). Yellow solid, yield 0.24 g (93%), m.p. 472–473 K, *R*_F_ = 0.38 (9.1% ethyl acetate–hep­ta­ne). IR (ATR, cm^−1^): 3026 [C(*sp* ^2^)H], 1663 (C=O), 1627 (C=N), 1589 (C=C_vin­yl_), 1537 (C=C_arom_), 1483 (C=C_arom_), 957 (=C—H_*trans*_). NMR (CDCl_3_): δ(^1^H) (400 MHz) 8.18 (*d*, *J* = 8.3 Hz, 1H, H8), 8.11 (*d*, *J* = 8.3 Hz, 1H, H5), 7.99 (*d*, *J* = 15.5 Hz, 1H, =CH_B′_), 7.82–7.78 (*m*, 1H, H7), 7.77–7.75 (*m*, 2H, H2′′, H6′′), 7.58 (*ddd*, *J* = 8.3, 6.8, 1.2 Hz, 1H, H6), 7.56–7.52 (*m*, 1H, H4′′), 7.43 (*d*, *J* = 8.4 Hz, 2H, H3′, H5′), 7.38 (*t*, *J* = 7.8 Hz, 2H, H3′′, H5′′), 7.33 (*d*, *J* = 8.4 Hz, 2H, H2′, H6′), 7.21 (*d*, *J* = 4.9 Hz, 1H, H3′′′), 7.08 (*d*, *J* = 15.5 Hz, 1H, H_A′_C=), 7.08 (*d*, *J* = 16.2 Hz, 1H, H_a′′′_C=), 6.99 (*d*, *J* = 16.2 Hz, 1H, =CH_B′′′_), 6.98–6.95 (*m*, 2H, H4′′′, H5′′′); δ(^13^C) (100 MHz) 198.5 (C=O), 151.4 (C2), 148.1 (C8a), 141.7 (C4), 141.3 (C2′′′), 137.8 (C1′′), 135.4 (C1′), 135.0 (=CH_B′_), 134.0 (C4′′), 132.3 (=CH_B′′′_), 131.8 (C3′, C5′), 130.9 (C3), 130.6 (C7), 130.0 (C8), 129.5 (C2′′, C6′′), 129.0 (C2′, C6′), 128.9 (C3′′, C5′′), 128.0 (C5′′′), 127.7 (C4′′′), 127.0 (C6), 126.1 (C3′′′), 125.2 (C4a), 125.1 (H_A′_C=), 124.9 (C5), 122.7 (C4′), 121.1 (H_A′′′_C=). HRMS (ESI^+^) *m*/*z* found for [*M* + H]^+^ 522.05249, C_30_H_20_^79^BrNOS = requires 522.05217.

### Refinement

Crystal data, data collection and refinement details for compounds (IIa)–(IIe) are summarized in Table 1 ▸. For compound (IId), the reflection 100, which had been attenuated by the beam stop, was removed from the data set. In addition, a small number of bad outlier reflections [ 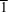 04 for (IIa) and 141, 231, 241, 033 and 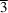 03 for (IIc)] were also removed. Compound (IIc) was handled as a nonmerohedral twin, with twin matrix (−0.053, 0.000, 0.947/0.000, −1.000, 0.000/1.053, 0.000, 0.053) and with refined twin fractions of 0.865 (2) and 0.135 (2). In compound (IIe), the thienyl unit was disordered over two sets of atomic sites having unequal occupancies. For the minor-disorder component, the bonded distances and the 1,3 nonbonded distances were restrained to be the same as the corresponding distances in the major-disorder component, subject to s.u. values of 0.01 and 0.02 Å, respectively. In addition, the anisotropic displacement parameters for pairs of partial-occupancy atoms occupying essentially the same physical space were constrained to be identical. All H atoms, apart from those in the minor-disorder component of compound (IIe), were located in difference maps and then treated as riding atoms in geometrically idealized positions, with C—H = 0.95 Å and *U*_iso_(H) = 1.2*U*_eq_(C); the H atoms in the minor-disorder component of compound (IIe) were included in the refinement in exactly the same manner. Subject to these conditions, the refined occupancy values for the disorder components of (IIe) were 0.926 (3) and 0.074 (3). In the final difference map, the largest maximum of 1.65 e Å^−3^ was 0.86 Å from atom Br24, while the largest minimum of −1.18 e Å^−3^ was 0.66 Å from Br24. While these features might indicate some further minor disorder, the anisotropic displacement parameters provided no support for this possibility, which was therefore not pursued further.

## Results and discussion

The 2-methyl-4-styryl­quino­line precursors of type (I) (see Scheme 1) were prepared in high yields using Friedländer annulation reactions (Meléndez *et al.*, 2020 ▸; Vera *et al.*, 2022 ▸) between (2-amino­phen­yl)chalcones and either acetone, for compounds (Ia) and (Ib), or 1-phenyl­butane-l-1,3-dione, for compounds (Ic)–(Ie). These precursors of type (I) were then converted successfully into the target 2,4-distyryl­quino­lines (IIa)–(IIe) in yields of 71–93% by means of indium tri­chloride-catalyzed Knoevenagel-type condensation reactions with the appropriate aromatic aldehydes (see Scheme 1). Compounds (IIa)–(IIe) were all fully characterized by standard spectroscopic means (FT–IR, ^1^H and ^13^C NMR spectroscopy, and high-resolution mass spectrometry) and full details of the spectroscopic characterization are provided in Section 2.1.

The formation of the second styryl fragment in products (IIa)–(IIe) was established by the disappearance from both the ^1^H and ^13^C NMR spectra of the signals from the methyl group at position C2, and their replacement by new sets of signals corresponding to the newly-introduced C and H atoms; thus, eight new C atoms in each case and seven new H atoms in (IIa), five in (IIb) and six in each of (IIc)–(IIe). In each case, the Knoevenagel-type condensation proceeded in a highly stereoselective manner giving exclusively the *E* stereoisomers, as indicated by the ^1^H NMR spectra. The *E* configuration of the newly-formed styryl fragment was deduced on the basis of the coupling constant values (^3^ *J*_HA′,HB′_ *ca* 16.0 Hz) between HA′ and HB′. The constitutions of compounds (IIa)–(IIe), which were deduced from the spectroscopic data, were then fully confirmed by the results of single-crystal X-ray diffraction, which additionally provided information on the mol­ecular conformations and the inter­molecular inter­actions in the solid state.

The versatility of this synthetic route to 2,4-distyryl­quino­lines and their analogues is underpinned by the possibility of incorporating a wide variety of substituents into the initial chalcone precursor, into the ketone employed in the annulation step and into the aldehyde used in the final condensation step.

In compound (IIe), the thio­phene unit is disordered over two sets of atomic sites having occupancies of 0.926 (3) and 0.074 (3), such that the two disorder forms are related by a rotation of approximately 180° around the exocyclic C—C bond; the dihedral angle between the mean planes of the two disorder components is only 4(2)°.

The mol­ecules of compounds (IIa)–(IIe) exhibit no inter­nal symmetry and hence these compounds are all conformationally chiral (Moss, 1996 ▸; Flack & Bernardinelli, 1999 ▸), but the centrosymmetric space groups (Table 1 ▸) confirm that equal numbers of the two conformational enanti­omers are present in each of (IIa)–(IIe).

**Figure 1:**
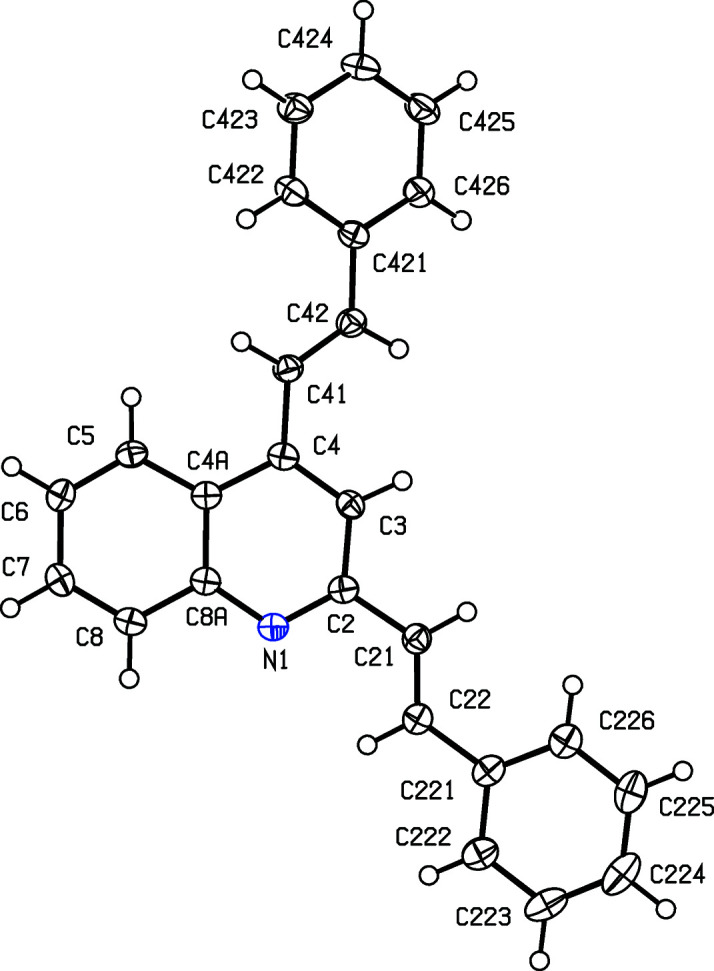
The mol­ecular structure of compound (IIa), showing the atom-labelling scheme. Displacement ellipsoids are drawn at the 50% probability level.

For the 3-benzoyl products (IIc)–(IIe), the reference mol­ecules are all such that the torsion angle C2—C3—C31—C311 has a positive sign (Table 2 ▸), while the value of the torsion angle C3—C4—C41—C42 in (IIc) is markedly different from those in the other four compound reported here (Table 2 ▸ and Figs. 1 ▸–5 ▸ ▸ ▸ ▸). In each of (IIa)–(IIe), the 2-styryl unit is close to being coplanar with the quino­line unit, while the 4-substituent is twisted well out of the plane of the quino­line unit. These observations thus complement the general pattern in styryl­quino­lines that we have noted previously (Vera *et al.*, 2022 ▸; Ardila *et al.*, 2022 ▸). Amongst the styryl­quino­lines whose structures are recorded in the Cambridge Structural Database (Groom *et al.*, 2016 ▸), 2-styryl- and 8-styryl­quino­lines all have mol­ecular skeletons which are effectively planar, while in 4-styryl­quino­lines, the styryl unit is always markedly twisted out of the plane of the quino­line unit by a rotation about the exocyclic bond corresponding to C4—C41 in the numbering system used here.

Despite this, there are some unexpected differences in the mol­ecular orientations of the two aryl­vinyl units (Figs. 1 ▸–5 ▸ ▸ ▸ ▸ and Table 2 ▸). Thus, the orientation of the 2-styryl substituent in (IIb) differs from that in each of (IIa) and (IIc)–(IIe) by a rotation about the C2—C21 bond of approximately 180°. In addition, the orientation of the 4-stryl unit in (IIc) differs markedly from that in each of the other examples, but the torsion angle C3—C4—C41—C42 shows quite a wide range of variation (Table 2 ▸). These differences in conformation cannot reasonably be explained in terms of the patterns of hy­dro­gen bonding discussed below (*cf*. Table 3 ▸).

**Figure 6:**
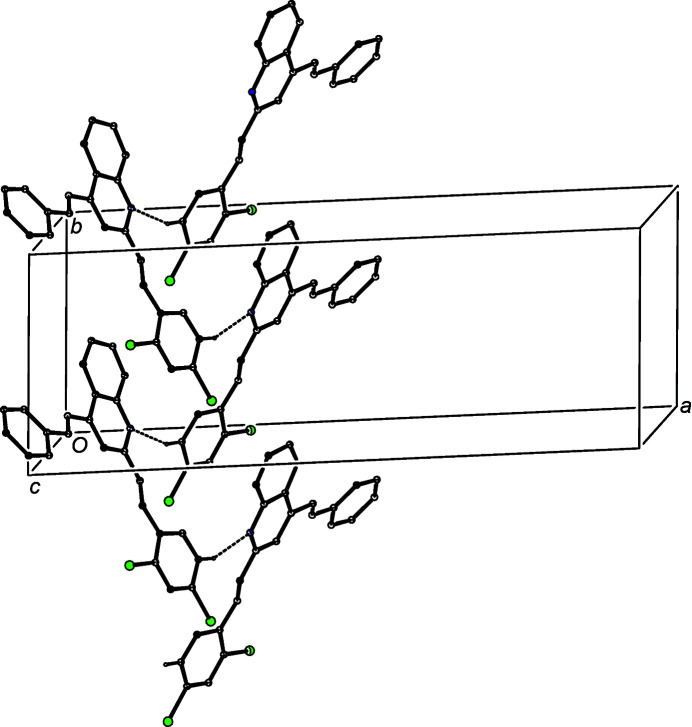
Part of the crystal structure of compound (IIb), showing the formation of a *C*(8) chain parallel to [010], built from C—H⋯N hy­dro­gen bonds, which are drawn as dashed lines. For the sake of clarity, H atoms which are not involved in the motif shown have been omitted.

**Figure 7:**
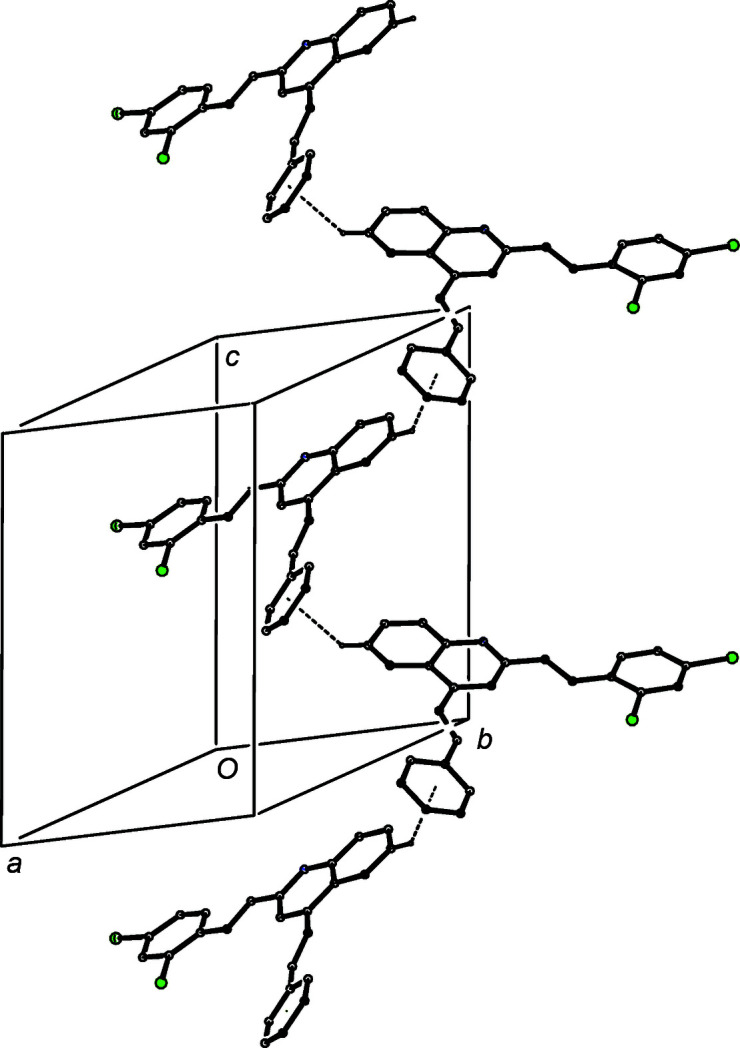
Part of the crystal structure of compound (IIb), showing the formation of a chain parallel to [001], built from C—H⋯π(arene) hy­dro­gen bonds, which are drawn as dashed lines. For the sake of clarity, H atoms which are not involved in the motif shown have been omitted.

**Figure 8:**
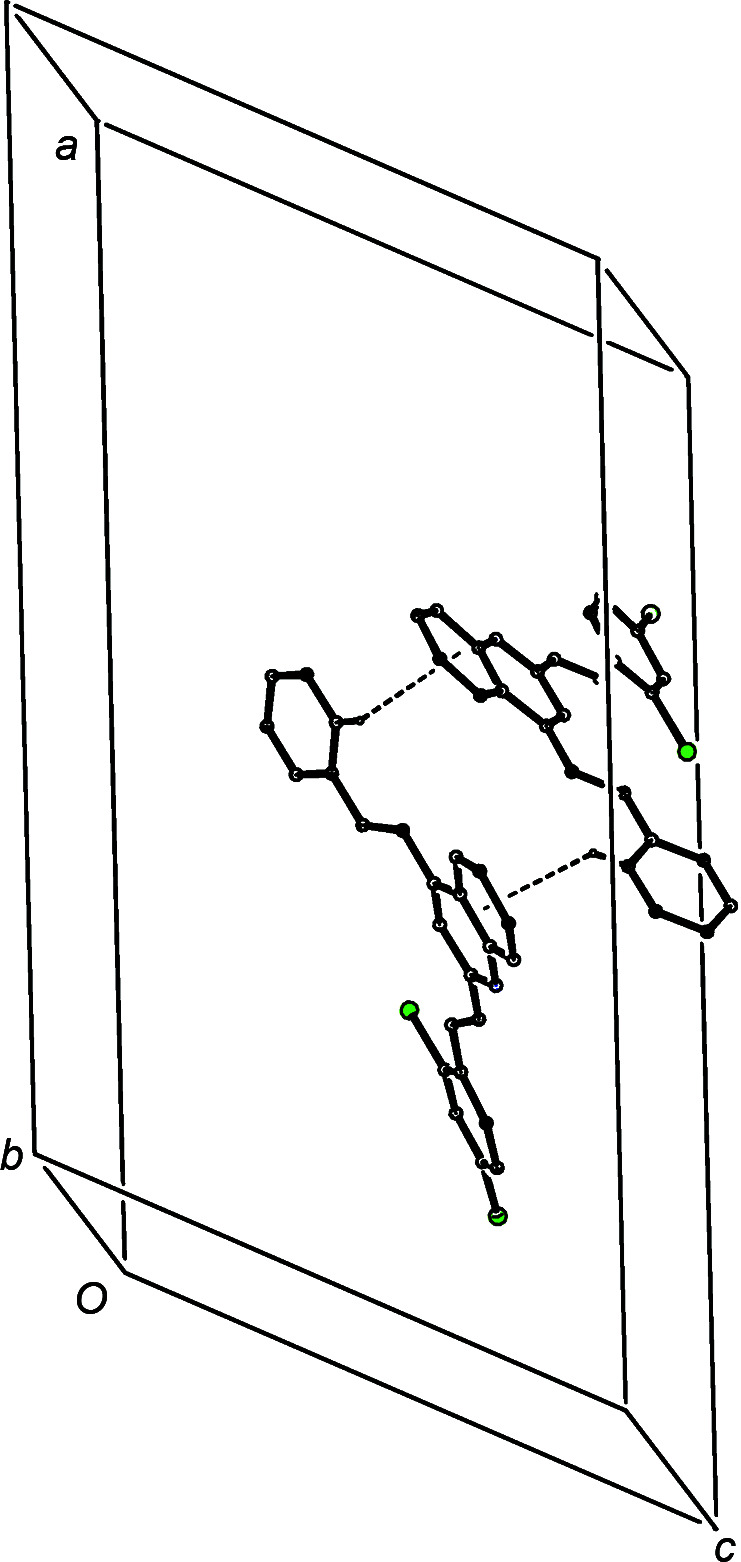
Part of the crystal structure of compound (IIb), showing the formation of a centrosymmetric dimer built from C—H⋯π(arene) hy­dro­gen bonds, which are drawn as dashed lines. For the sake of clarity, H atoms which are not involved in the motif shown have been omitted.

The patterns of supra­molecular assembly in compounds (IIa)–(IIe) show some wide variations. Despite the large numbers of aromatic rings and C—H bonds in the mol­ecules of (IIa), the crystal structure contains no significant direction-specific inter­molecular inter­actions of any sort. By contrast, in the di­chloro analogue (IIb), a combination of one C—H⋯N hy­dro­gen bond and two independent C—H⋯π(arene) hy­dro­gen bonds (Table 3 ▸) links the mol­ecules into a three-dimensional framework structure, whose formation is readily analysed in terms of three simple substructures (Ferguson *et al.*, 1998*a* ▸,*b* ▸; Gregson *et al.*, 2000 ▸). The C—H⋯N hy­dro­gen bonds link mol­ecules of (IIb) which are related by the 2_1_ screw axis along ( 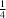 , *y*, 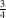 ) to form a *C*(8) (Etter, 1990 ▸; Etter *et al.*, 1990 ▸; Bernstein *et al.*, 1995 ▸) chain running parallel to the [010] direction (Fig. 6 ▸). In the second substructure, the C—H⋯π(arene) hy­dro­gen bond having atom C6 as the donor links mol­ecules which are related by the *c*-glide plane at *y* = 1 to form a chain running parallel to the [001] direction (Fig. 7 ▸). The combination of the chains along [010] and [001] generates a sheet lying parallel to (100) in the domain 0 < *x* < 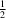 . A second sheet, related to the first by inversion, lies in the domain 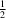 < *x* < 1.0, and adjacent sheets are linked by the third substructure which takes the form of a cyclic centrosymmetric dimer built from C—H⋯π(arene) hy­dro­gen bonds having atom C422 as the donor (Fig. 8 ▸).

**Figure 9:**
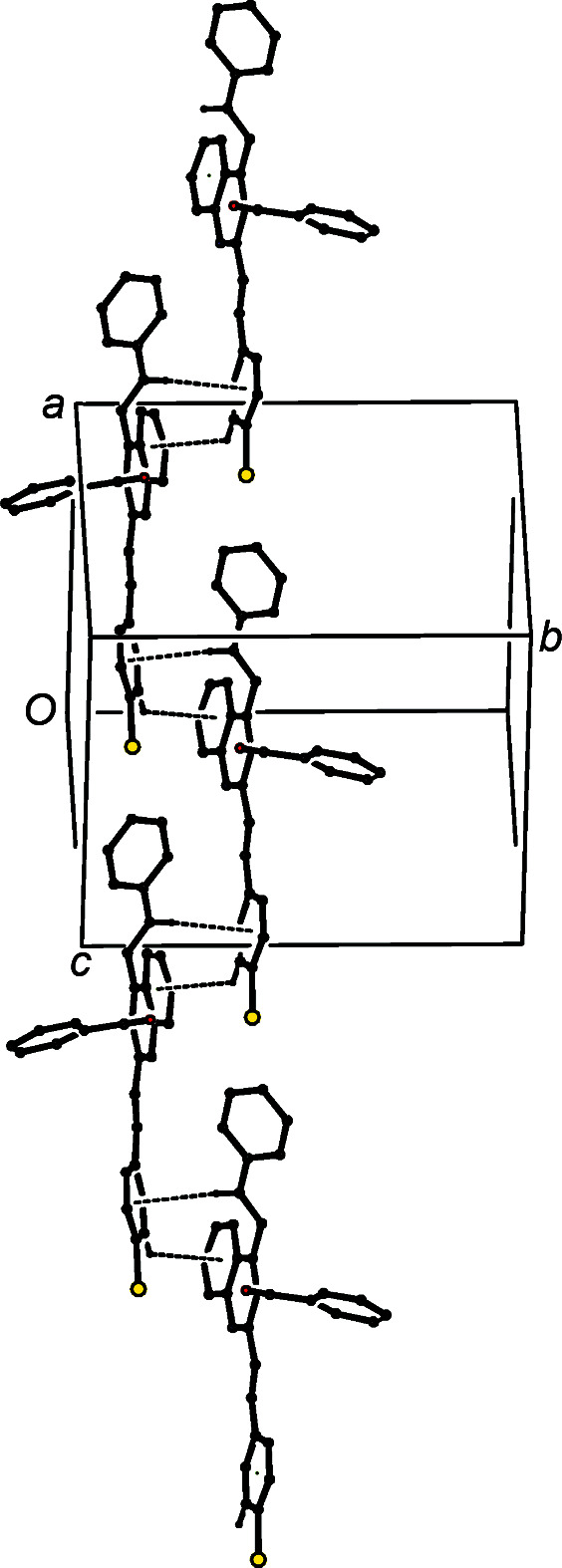
Part of the crystal structure of compound (IIc), showing the formation of a chain of rings running parallel to the [10 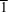 ] direction and built from two independent C—H⋯π(arene) hy­dro­gen bonds, which are drawn as dashed lines. For the sake of clarity, H atoms which are not involved in the motif shown have been omitted.

The short inter­molecular C—H⋯O contact in compound (IIc) has a very small C—H⋯O angle (Table 3 ▸), and so cannot be regarded as structurally significant (Wood *et al.*, 2009 ▸). However, the co-operative action of two C—H⋯π(arene) hy­dro­gen bonds links mol­ecules which are related by the *n*-glide plane at *y* = 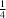 into a chain of rings running parallel to the [10 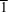 ] direction (Fig. 9 ▸). The structure also contains a third C—H⋯π(arene) contact, involving atom C5, but here the H⋯*A* distance is quite long; if this were regarded as structurally significant, its action would be to link the chains of rings into a sheet parallel to (101). 
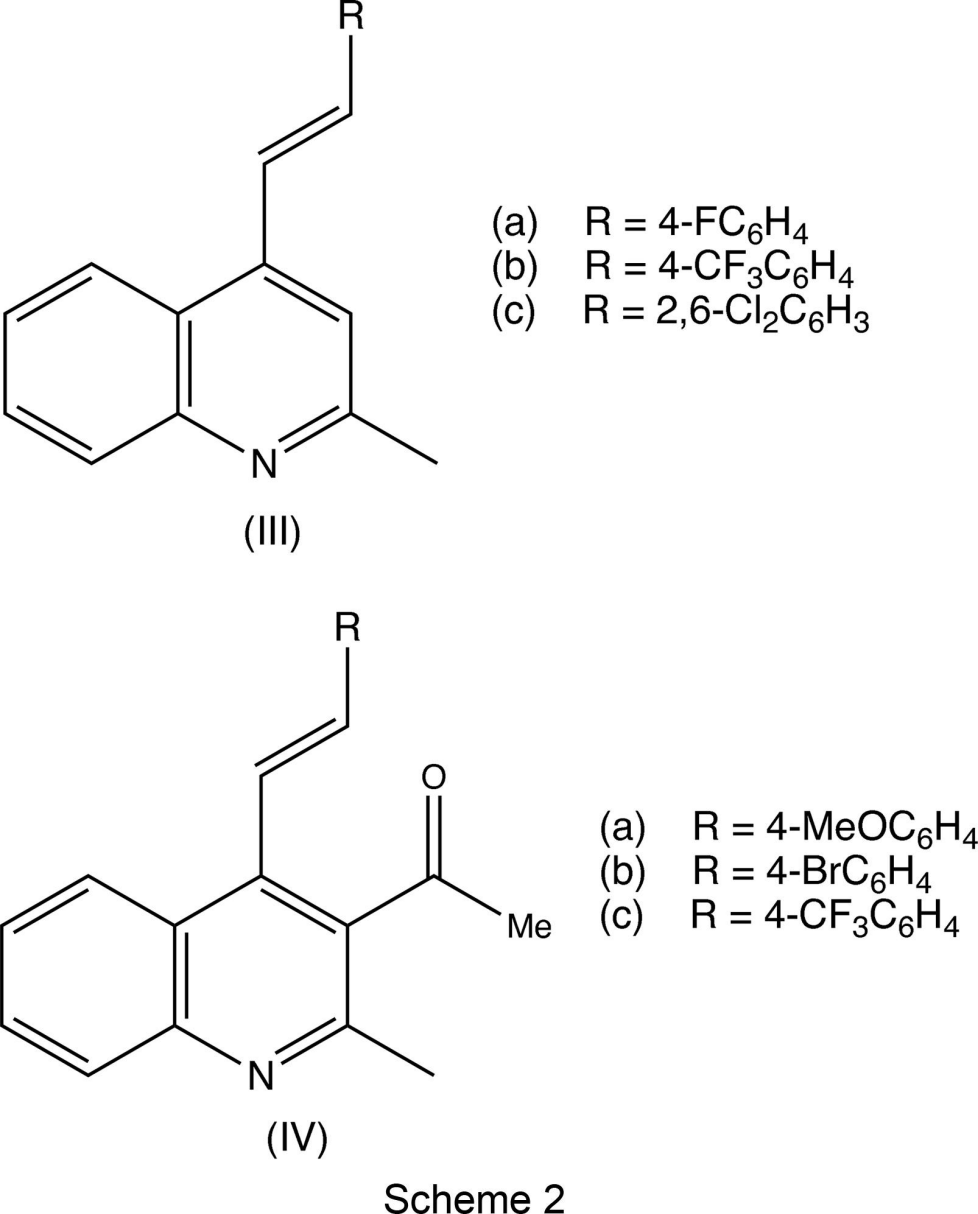


**Figure 10:**
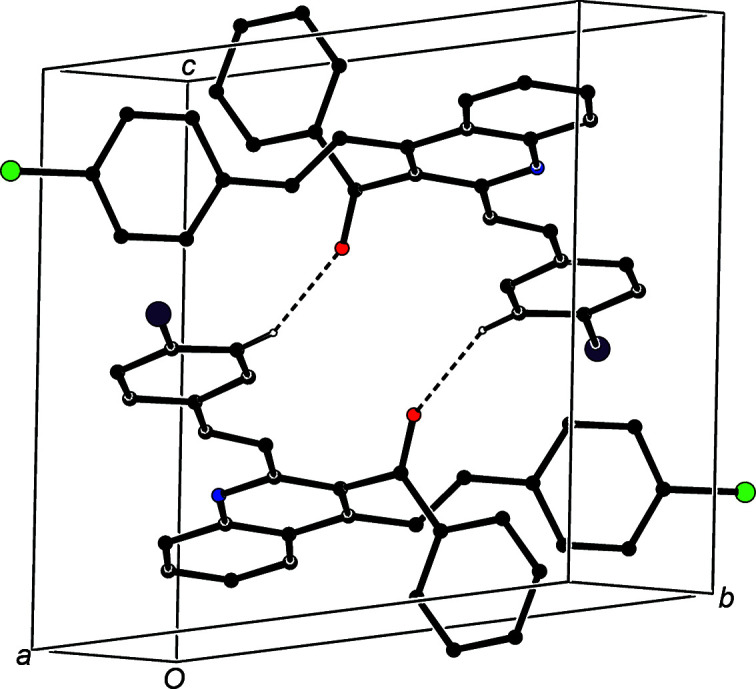
Part of the crystal structure of compound (IId), showing the formation of a cyclic 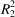 (20) dimer built from C—H⋯O hy­dro­gen bonds, which are drawn as dashed lines. For the sake of clarity, H atoms which are not involved in the motif shown have been omitted.

A single C—H⋯O hy­dro­gen bond links inversion-related mol­ecules of compound (IId) into a cyclic centrosymmetric 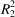 (20) dimer (Fig. 10 ▸), but there are no direction-specific inter­actions between adjacent dimers.

**Figure 11:**
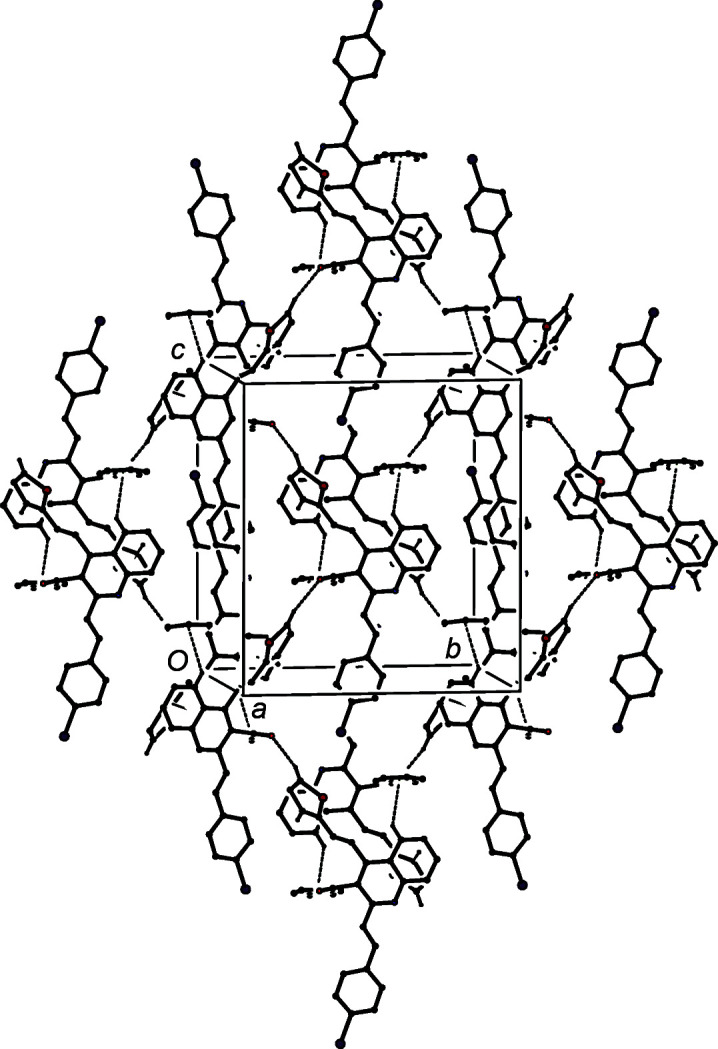
Part of the crystal structure of compound (IIe), showing the formation of a sheet lying parallel to {100} and built from a combination of C—H⋯O and C—H⋯π(arene) hy­dro­gen bonds, which are drawn as dashed lines. For the sake of clarity, the minor-disorder component and H atoms which are not involved in the motif shown have been omitted.

In the crystal structure of compound (IIe), the C—H⋯O contact involving atom C8 is not structurally significant (Wood *et al.*, 2009 ▸), but the combination of the C—H⋯O hy­dro­gen bond involving atom C425 with the two C—H⋯π(arene) hy­dro­gen bonds links the mol­ecules into a complex sheet lying parallel to (100) in the domain 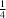 < *x* < 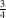 (Fig. 11 ▸). A second sheet, related to the first by the action of the 2_1_ screw axes, lies in the domain 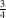 < *x* < 1.35, but there are no direction-specific inter­actions between adjacent sheets.

It is of inter­est briefly to compare the supra­molecular assembly in compounds (IIa)–(IIe) reported here with those of some simpler 2-methyl-4-styryl­quino­line analogues. Crystal structures have been reported (Vera *et al.*, 2022 ▸) for compounds (IIIa)–(IIIc) (see Scheme 2), which have no sub­sti­tuent at position C3 of the quino­line unit, and are thus related to compounds (IIa) and (IIb) reported here. In the crystal structure of (IIIa), the mol­ecules are linked into sheets by a combination of C—H⋯N hy­dro­gen bonds and π–π stacking inter­actions, while a similar combination of inter­actions links the mol­ecules of (IIIb) into chains of rings. There are no hy­dro­gen bonds in the structure of (IIIc), but a π–π stacking inter­action links the mol­ecules into stacks.

Compounds of the type (IV) (see Scheme 2), carrying a 3-acetyl substituent, are thus analogous to compounds (IIc)–(IIe). Compounds (IVa)–(IVc) are isomorphous (Rod­ríguez *et al.*, 2020 ▸); in each, the mol­ecules are linked into chains by a C—H⋯O hy­dro­gen bond, but only in (IVa) is this augmented by a C—H⋯π hy­dro­gen bonds to form a chain of rings. Thus, although (IVa)–(IVc) are isomorphous, they are not strictly isostructural.

## Summary

We have developed an efficient and highly versatile route to 2,4-distyryl­quino­lines and to their 2-aryl­vin­yl analogues, using only simple and readily accessible building blocks such as simple aldehydes and ketone. We have characterized by spectroscopic means (IR, ^1^H and ^13^C NMR spectroscopy, and HRMS) five representative examples and we have determined their mol­ecular and crystal structures, which fully confirm the mol­ecular constitutions deduced from the spectroscopic data, as well as providing further information on their mol­ecular conformations in the solid state, and on their supra­molecular assemblies.

## Supplementary Material

Crystal structure: contains datablock(s) global, IIa, IIb, IIc, IId, IIe. DOI: 10.1107/S2053229623001432/ov3167sup1.cif

Structure factors: contains datablock(s) IIa. DOI: 10.1107/S2053229623001432/ov3167IIasup2.hkl

Structure factors: contains datablock(s) IIb. DOI: 10.1107/S2053229623001432/ov3167IIbsup3.hkl

Structure factors: contains datablock(s) IIc. DOI: 10.1107/S2053229623001432/ov3167IIcsup4.hkl

Structure factors: contains datablock(s) IId. DOI: 10.1107/S2053229623001432/ov3167IIdsup5.hkl

Structure factors: contains datablock(s) IIe. DOI: 10.1107/S2053229623001432/ov3167IIesup6.hkl

Supporting information file. DOI: 10.1107/S2053229623001432/ov3167IIasup7.cml

Supporting information file. DOI: 10.1107/S2053229623001432/ov3167IIbsup8.cml

Supporting information file. DOI: 10.1107/S2053229623001432/ov3167IIcsup9.cml

Supporting information file. DOI: 10.1107/S2053229623001432/ov3167IIdsup10.cml

Supporting information file. DOI: 10.1107/S2053229623001432/ov3167IIesup11.cml

CCDC references: 2242537, 2242536, 2242535, 2242534, 2242533

## Figures and Tables

**Figure 2:**
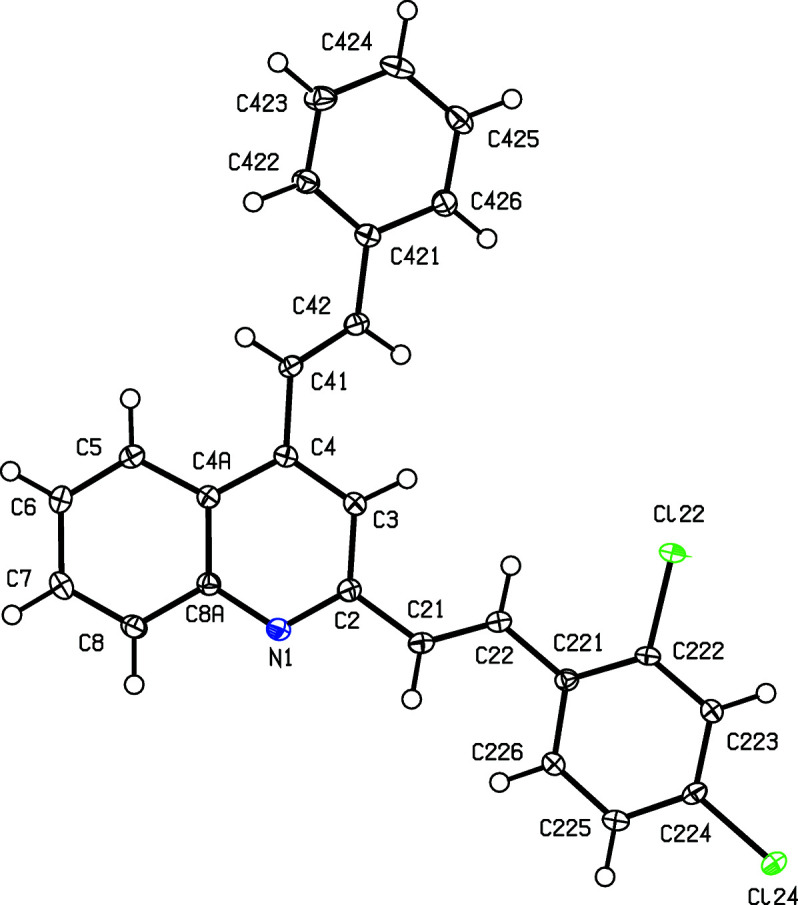
The mol­ecular structure of compound (IIb), showing the atom-labelling scheme. Displacement ellipsoids are drawn at the 50% probability level.

**Figure 3:**
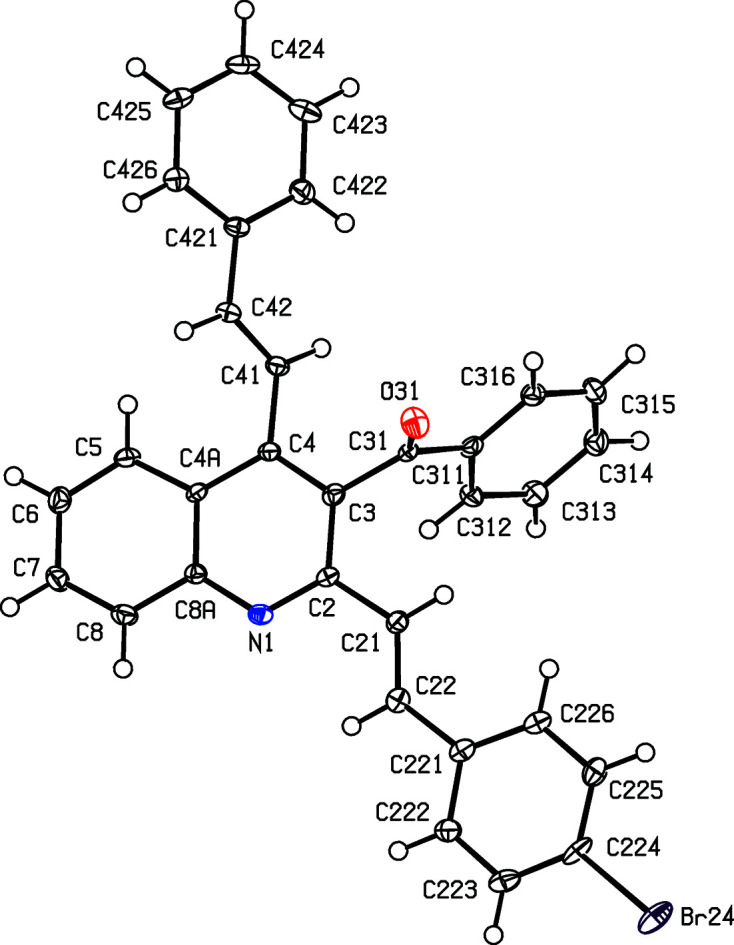
The mol­ecular structure of compound (IIc), showing the atom-labelling scheme. Displacement ellipsoids are drawn at the 50% probability level.

**Figure 4:**
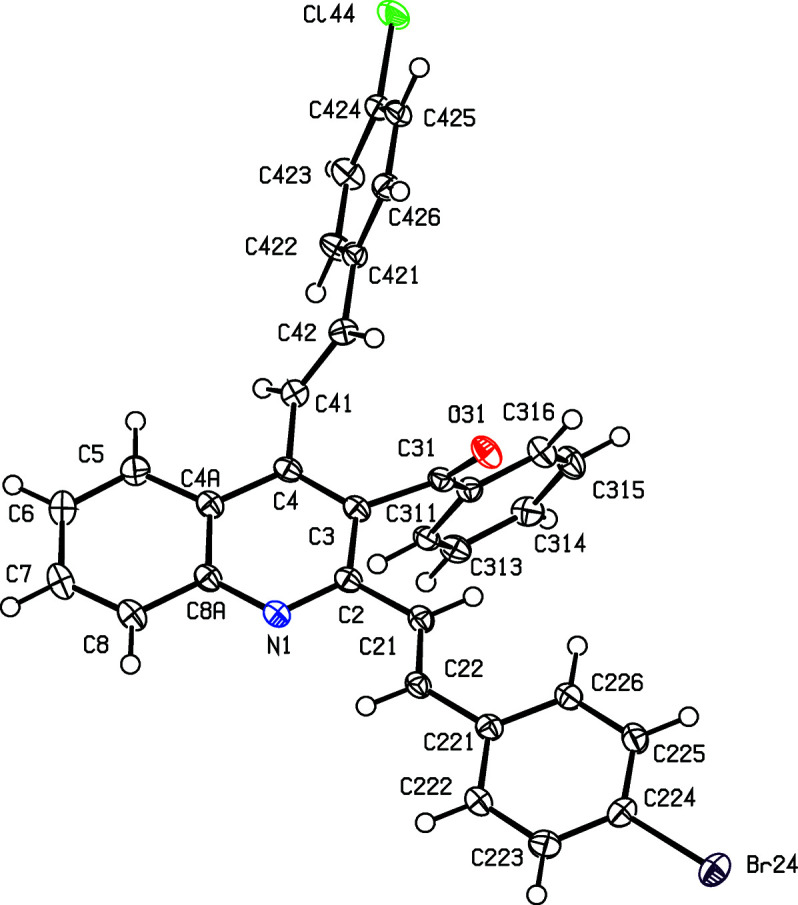
The mol­ecular structure of compound (IId), showing the atom-labelling scheme. Displacement ellipsoids are drawn at the 50% probability level.

**Figure 5:**
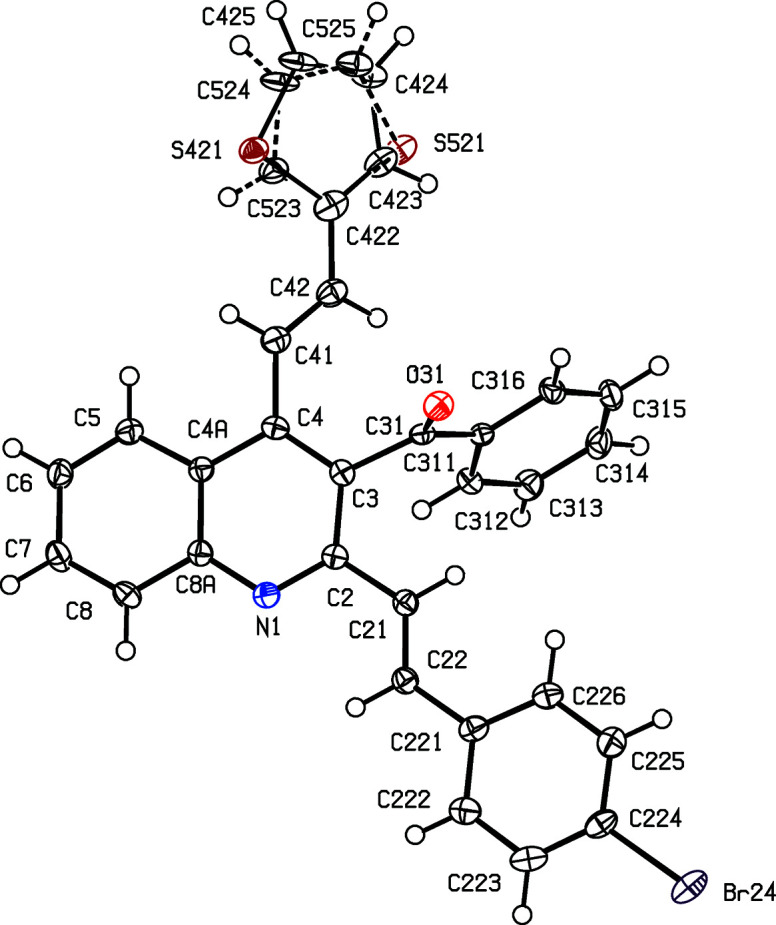
The mol­ecular structure of compound (IIe), showing the conformational disorder and the atom-labelling scheme. The major-disorder component is drawn with full lines and the minor-disorder component is drawn using broken lines. Displacement ellipsoids are drawn at the 50% probability level.

**Table:** Experiments were carried out at 100 K with Mo *K*α radiation using a Bruker D8 Venture diffractometer. Absorption was corrected for by multi-scan methods (*SADABS*; Bruker, 2016 ▸). H-atom parameters were constrained.

	(IIa)	(IIb)	(IIc)
Crystal data			
Chemical formula	C_25_H_19_N	C_25_H_17_Cl_2_N	C_32_H_22_BrNO
*M*_r_	333.41	402.29	516.41
Crystal system, space group	Monoclinic, *P*2_1_/*c*	Monoclinic, *C*2/*c*	Monoclinic, *P*2_1_/*n*
*a*, *b*, *c* (Å)	12.6112 (6), 8.6352 (4), 17.3080 (8)	28.4950 (7), 9.5384 (3), 16.0520 (5)	12.287 (2), 15.528 (3), 12.844 (3)
α, β, γ (°)	90, 105.925 (2), 90	90, 118.581 (1), 90	90, 99.877 (6), 90
*V* (Å^3^)	1812.51 (15)	3831.2 (2)	2414.2 (8)
*Z*	4	8	4
μ (mm^−1^)	0.07	0.35	1.73
Crystal size (mm)	0.19 × 0.14 × 0.08	0.21 × 0.10 × 0.09	0.15 × 0.12 × 0.08

Data collection			
*T*_min_, *T*_max_	0.924, 0.994	0.901, 0.969	0.720, 0.871
No. of measured, independent and observed [*I* > 2σ(*I*)] reflections	38720, 4161, 3388	47555, 4414, 3914	5530, 5530, 4290
*R*_int_	0.060	0.056	–
(sin θ/λ)_max_ (Å^−1^)	0.650	0.650	0.653

Refinement			
*R*[*F* ^2^ > 2σ(*F* ^2^)], *wR*(*F* ^2^), *S*	0.050, 0.118, 1.09	0.032, 0.078, 1.09	0.064, 0.196, 1.05
No. of reflections	4161	4414	5530
No. of parameters	235	253	317
No. of restraints	0	0	0
Δρ_max_, Δρ_min_ (e Å^−3^)	0.25, −0.24	0.35, −0.22	1.40, −0.70

**Table:** 

	(IId)	(IIe)
Crystal data		
Chemical formula	C_32_H_21_BrClNO	C_30_H_20_BrNOS
*M*_r_	550.86	522.44
Crystal system, space group	Triclinic, *P* 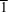	Orthorhombic, *P* *b* *c* *a*
*a*, *b*, *c* (Å)	9.9051 (12), 11.3936 (16), 11.8192 (16)	15.5785 (8), 16.4215 (7), 18.3126 (9)
α, β, γ (°)	77.727 (5), 76.116 (5), 86.448 (5)	90, 90, 90
*V* (Å^3^)	1265.2 (3)	4684.8 (4)
*Z*	2	8
μ (mm^−1^)	1.76	1.87
Crystal size (mm)	0.19 × 0.12 × 0.10	0.20 × 0.12 × 0.08

Data collection		
*T*_min_, *T*_max_	0.735, 0.836	0.768, 0.861
No. of measured, independent and observed [*I* > 2σ(*I*)] reflections	61838, 5807, 5014	63672, 5363, 4456
*R*_int_	0.064	0.060
(sin θ/λ)_max_ (Å^−1^)	0.650	0.649

Refinement		
*R*[*F* ^2^ > 2σ(*F* ^2^)], *wR*(*F* ^2^), *S*	0.028, 0.065, 1.03	0.040, 0.101, 1.02
No. of reflections	5807	5363
No. of parameters	325	320
No. of restraints	0	10
Δρ_max_, Δρ_min_ (e Å^−3^)	0.33, −0.39	1.65, −1.18

Computer programs: *APEX3* (Bruker, 2018 ▸), *SAINT* (Bruker, 2017 ▸), *SHELXT2014* (Sheldrick, 2015*a* ▸), *SHELXL2014* (Sheldrick, 2015*b* ▸) and *PLATON* (Spek, 2020 ▸).

**Table 2:** Selected torsion angles (°) for compounds (IIa)–(IIe)

Parameter	(IIa)	(IIb)	(IIc)	(IId)	(IIe)
C3—C2—C21—C22	−178.77 (14)	−12.9 (2)	−171.8 (4)	179.16 (16)	178.5 (2)
C21—C22—C221—*C*222	−173.99 (15)	−161.58 (14)	171.4 (4)	−171.95 (17)	−174.5 (3)
C2—C3—C31—C311			87.2 (5)	109.43 (17)	85.9 (3)
C3—C31—C311—C312			−15.6 (6)	−12.3 (2)	−10.9 (3)
C3—C4—C41—C42	19.8 (2)	23.4 (2)	−131.9 (4)	−44.4 (2)	37.6 (4)
C41—C42—C421—C422	8.3 (2)	−6.1 (2)	−4.5 (6)	−21.4 (3)	
C41—C42—C422—S421					0.8 (4)
C41—C42—C522—S521					−175.9 (7)

**Table 3:** Hydrogen bonds and short inter­molecular contacts (Å, °) for compounds (IIb)–(IIe) *Cg*1–*Cg*4 represent the centroids of rings C421–C426, C4*A*/C5–C8/C8*A*, C311–C316 and C221–C216, respectively.

Compound	*D*—H⋯*A*		*D*—H	H⋯*A*	*D*⋯*A*	*D*—H⋯*A*
(IIb)	C225—H225⋯N1^i^		0.95	2.62	3.522 (2)	158
	C6—H6⋯*Cg*1^ii^		0.95	2.65	3.4152 (16)	138
	C422—H422⋯*Cg*2^iii^		0.95	2.88	3.5843 (17)	132
(IIc)	C8—H8⋯O31^iv^		0.95	2.51	3.164 (6)	126
	C5—H5⋯*Cg*3^v^		0.95	2.96	3.728 (4)	138
	C42—H42⋯*Cg*4^vi^		0.95	2.88	3.766 (5)	155
	C223—H223⋯*Cg*2^vii^		0.95	2.84	3.429 (3)	122
(IId)	C225—H225⋯O31^v^		0.95	2.37	3.266 (2)	156
(IIe)	C8—H8⋯O31^viii^		0.95	2.58	3.122 (3)	116
	C425—H425⋯O31^ix^		0.95	2.45	3.361 (4)	161
	C5—H5⋯*Cg*3^v^		0.95	2.90	3.647 (3)	136
	C423—H423⋯*Cg*3^v^		0.95	2.76	3.449 (3)	130

Symmetry codes: (i) −*x* + 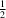 , *y* − 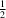 , −*z* + 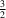 ; (ii) *x*, −*y* + 2, *z* + 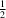 ; (iii) −*x* + 1, *y*, −*z* + 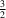 ; (iv) *x* + 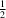 , −*y* + 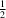 , *z* + 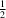 ; (v) −*x* + 1, −*y* + 1, −*z* + 1; (vi) *x* + 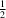 , −*y* + 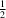 , *z* − 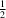 ; (vii) *x* − 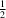 , −*y* + 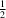 , *z* + 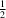 ; (viii) −*x* + 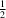 , *y* − 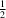 , *z*; (ix) *x*, −*y* + 2, *z* − 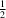 .
